# Variability in usual care fluid resuscitation and risk-adjusted outcomes for mechanically ventilated patients in shock

**DOI:** 10.1186/s13054-020-2734-9

**Published:** 2020-01-28

**Authors:** Jason N. Mansoori, Walter Linde-Zwirble, Peter C. Hou, Edward P. Havranek, Ivor S. Douglas

**Affiliations:** 1 Division of Pulmonary, Critical Care and Sleep Medicine, Department of Medicine, Denver Health Medical Center, 601 Broadway, MC 4000, Denver, CO 80203 USA; 2 Division of Pulmonary Sciences and Critical Care Medicine, Department of Medicine, University of Colorado School of Medicine, Aurora, USA; 3 Trexin Consulting, Minneapolis, USA; 4 Division of Emergency Care Medicine, Department of Emergency Medicine, Brigham and Women’s Hospital, Harvard Medical School, Boston, USA; 5 Division of Cardiology, Department of Medicine, Denver Health Medical Center, Denver, USA; 6 Division of Cardiology, Department of Medicine, University of Colorado School of Medicine, Aurora, USA

**Keywords:** Critical care outcomes, Critical care/utilization, Circulatory collapse, Fluid therapy/mortality, Clinical decision-making

## Abstract

**Rationale:**

There remains significant controversy regarding the optimal approach to fluid resuscitation for patients in shock. The magnitude of care variability in shock resuscitation, the confounding effects of disease severity and comorbidity, and the relative impact on sepsis survival are poorly understood.

**Objective:**

To evaluate usual care variability and determine the differential effect of observed and predicted fluid resuscitation volumes on risk-adjusted hospital mortality for mechanically ventilated patients in shock.

**Methods:**

We performed a retrospective outcome analysis of mechanically ventilated patients admitted to intensive care units using the 2013 Premier Hospital Database (Premier, Inc.). Observed and predicted hospital mortality were evaluated by observed and predicted day 1 fluid administration, using the difference in predicted and observed outcomes to adjust for disease severity between groups. Both predictive models were validated using a second large administrative database (Truven Health Analytics Inc.). Secondary outcomes included duration of mechanical ventilation, hospital and ICU length of stay, and cost.

**Results:**

Among 33,831 patients, observed hospital mortality was incrementally higher than predicted for each additional liter of day 1 fluid beginning at 7 L (40.9% vs. 37.2%, *p* = 0.008). Compared to patients that received expected (± 1.5 L predicted) day 1 fluid volumes, greater-than-expected fluid resuscitation was associated with increased risk-adjusted hospital mortality (52.3% vs. 45.0%, *p* < 0.0001) among all patients with shock and among a subgroup of shock patients with comorbid conditions predictive of lower fluid volume administration (47.1% vs. 41.5%, *p* < 0.0001). However, in patients with shock but without such conditions, both greater-than-expected (57.5% vs. 49.2%, *p* < 0.0001) and less-than-expected (52.1% vs. 49.2%, *p* = 0.037) day 1 fluid resuscitation were associated with increased risk-adjusted hospital mortality.

**Conclusions:**

Highly variable day 1 fluid resuscitation was associated with a non-uniform impact on risk-adjusted hospital mortality among distinct subgroups of mechanically ventilated patients with shock. These findings support closer evaluation of fluid resuscitation strategies that include broadly applied fluid volume targets in the early phase of shock resuscitation.

## Introduction

In the early phases of resuscitation for undifferentiated shock, uncertainty regarding the underlying etiology and patient comorbidities are common reasons for non-patient-centered resuscitation practices, conflicting treatments, unwarranted practice variability, and failure to meet targeted end points for initial resuscitation [1]. A positive association between fluid balance and mortality has been demonstrated in observational studies [2–8]. In smaller retrospective studies, however, comparisons of administered fluid volumes between resuscitation groups are frequently confounded by disease severity. Additionally, the extent and impact of variable fluid resuscitation decisions in common usual care practice is poorly characterized. Consequently, assessments of actual administered fluid volumes alone may not effectively address the clinical appropriateness of fluid resuscitation or the effects of relative under- and over-resuscitation on patient outcomes.

In a novel attempt to both characterize the nature and frequency of variable fluid administration practices in the initial phase of shock resuscitation and to adjust for confounding by disease severity, we conducted a retrospective outcome analysis of day 1 fluid (DOF) resuscitation in high-risk mechanically ventilated adult patients resuscitated for shock. We sought to determine the impact of variable DOF volumes on hospital mortality in the most severely ill patients, including those with undifferentiated shock and septic shock. Additionally, we sought to identify hospital- and patient-level contextual factors that contribute to irregular practice and evaluate whether these predict or explain differences in prescribed fluid volumes and hospital mortality. We hypothesized that mechanically ventilated patients with shock are frequently prescribed greater- or less-than-expected DOF volumes resulting in adverse impacts on standardized hospital mortality after risk adjustment.

## Methods

### Data source and study cohort

**Fig. 1:**
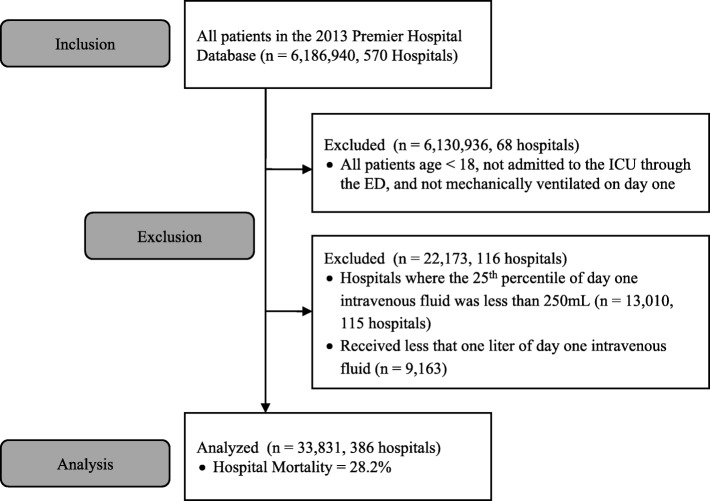
Patient and hospital selection. The 2013 Premier Hospital Database includes hospital discharges from January 1, 2013, to December 31, 2013. *ICU* intensive care unit, *ED* emergency department

De-identified data were obtained from the Premier Hospital Database (Premier Inc., Charlotte, NC, USA) and MarketScan Hospital Drug Database (Truven Health Analytics Inc., Ann Arbor, MI, USA). The study cohort was constructed using data from discharges in 2013 (Premier) and 2013–2016 (Truven). Both databases are comprised of highly detailed administrative billing data. Further details regarding each database are presented in the online supplement (see Additional file 1). Selection criteria were 18 years of age and older, medical patients, admitted through the emergency department, and admitted to the intensive care unit (ICU) with mechanical ventilation on hospital day 1. Hospitals where the 25th percentile for recorded DOF was below 250 mL were excluded to account for non-uniform level of detail documented between institutions. Since clock time is not recorded, fluid use was captured for the calendar day and not for a 24-h period. To compensate for this, cases with less than 1 L of DOF were excluded (Fig. 1). The study was exempt from Institutional Review Board oversight.

### Definitions

Patient data were arranged by the presence of a circulatory principal diagnosis, sepsis (ICD-9-CM codes for infection plus the use of parenteral antibiotics on admission), and shock (use of parenteral vasopressors on day 1 or day 2) [9]. For mechanical ventilation, we used daily itemized procedure billing data as opposed to ICD-9-CM codes, facilitating precise coding for each incremental day of mechanical ventilation. Patients were further characterized by Charlson-Deyo [10] comorbidities and by acute organ dysfunctions present at admission [9]. Hospitals were characterized by size (< 200, 200–399, 400+ beds), teaching status, urban/rural location, and US geographic region (Midwest, Northeast, South, and West). Definition codes and case mix adjustment are included and further described in the online supplement (see Additional file 1).

### Statistical analysis

The primary outcome was hospital mortality defined as all-cause mortality during the initial hospitalization. A predicted hospital mortality model using a generalized logistic link function and a linear ANOVA model for predicted DOF resuscitation were developed using the Premier Hospital Database and validated using the Truven MarketScan Hospital Drug Database. Both models included covariates for age, gender, over 200 ICD-9-CM admission diagnoses codes present in at least 3% of patients, and segments for patients with a principle circulatory or infectious diagnosis with or without shock. Further details regarding model development are presented in the online supplement (see Additional file 1).

Observed and predicted hospital mortality were then compared by DOF, categorized into 1-L increments up to 9 or more liters. In the absence of consensus definitions for under- or over-resuscitation, differences between those within an arbitrary but clinically relevant cutoff of ± 1.5 L from predicted DOF (“expected”) and those with greater- or less-than-expected DOF were also examined. Disease severity was adjusted for by adding the difference in predicted hospital mortality between expected and greater- or less-than-expected resuscitation groups to their respective observed hospital mortality. Repeated analyses in patient subgroups with sepsis and septic shock were also performed.

Secondary outcomes included total DOF resuscitation volumes, hospital length of stay (LOS), ICU LOS, duration of mechanical ventilation, and total cost. Lastly, DOF was compared by key hospital characteristics. Continuous variables are reported as means (±SD) or medians and interquartile ranges (IQR). Model covariates are reported as coefficients (95% confidence interval (CI), *p* value).

## Results

### Patients

Thirty-three thousand eight hundred thirty-one of 6,186,940 (0.55%) patients who met the inclusion and exclusion criteria and were discharged from 386 of 570 (67.7%) hospitals were analyzed (Fig. 1). The majority of patients were admitted to hospitals with 400 or more beds (44.3%), were non-teaching status (62.7%), urban (86.8%), and in the South region of the USA (52.3%) (Table 1).

**Table 1:** Cohort characteristics and secondary outcomes

	w/ FRF	w/o FRF	Less-than-expected*	Expected^†^	Greater-than-expected^‡^	All
Count, no. (%)	16,805 (49.7)	17,026 (50.3)	8502 (25.1)	18,379 (54.3)	6950 (20.5)	33,831
Count with shock, no. (%)	7967 (47.4)	7003 (41.1)	5033 (59.2)	6284 (34.2)	3653 (52.6)	14,970 (44.2)
Age, mean ± SD, years	66.6 ± 14.2	54.8 ± 18.8	59.6 ± 17.8	61.3 ± 17.6	60.5 ± 17.6	60.7 ± 17.7
Male gender, %	50.6	54.5	55.0	50.9	54.1	52.6
Race, %						
White	63.6	65.6	63.6	65.6	63.2	64.6
Black	18.7	14.8	16.8	17.0	16.0	16.8
Other	17.7	19.6	19.6	17.4	20.7	18.7
Comorbidities^§^, %						
Diabetes	41.2	22.5	30.5	32.1	32.6	31.8
CHF	57.3	0.0	25.3	30.3	27.4	28.5
Chronic pulmonary disease	15.9	11.4	13.9	13.6	13.4	13.7
Cerebrovascular disease	0.1	0.1	0.1	0.1	0.1	0.1
Dementia	1.1	0.8	1.0	1.0	0.9	1.0
Non-metastatic neoplasm	6.1	7.0	7.0	6.0	7.3	6.5
Metastatic neoplasm	2.4	3.6	3.3	2.8	3.0	3.0
Para and quadriplegia	2.4	2.6	2.0	2.9	2.1	2.5
Peripheral vascular disease	8.8	2.9	5.8	6.0	5.6	5.9
Rheumatic disease	3.3	2.1	2.6	2.8	2.6	2.7
Chronic renal disease	39.9	2.5	20.7	21.4	20.7	21.1
Mild liver disease	3.6	5.5	5.0	3.8	5.8	4.5
Major liver disease	2.5	4.9	4.1	2.9	5.4	3.7
Admission organ dysfunctions^§^, %						
Respiratory	100.0	100.0	100.0	100.0	100.0	100.0
Hematologic	11.8	11.9	15.5	9.3	14.1	11.9
CNS	25.7	24.6	27.9	23.1	27.0	25.1
Cardiac	47.4	41.1	59.2	34.2	52.6	44.2
Renal	38.8	28.5	41.3	28.0	39.1	33.6
Hepatic	4.6	4.8	6.6	3.4	5.9	4.7
Average per case, no.	2.3	2.1	2.5	2.0	2.4	2.2
Secondary outcomes^¶^						
Hospital LOS	7 (4–12)	4 (2–9)	5 (2–10)	6 (3–10)	6 (2–11)	6 (2–10)
ICU LOS	4 (2–7)	2 (1–5)	3 (1–6)	3 (2–6)	3 (2–7)	3 (2–6)
Length of MV	3 (2–7)	2 (2–5)	3 (2–6)	3 (2–5)	3 (2–6)	3 (2–6)
Cost in thousands, $	18.5 (10.8–32.2)	12.7 (7.2–24.0)	15.0 (7.9–27.9)	15.0 (8.5–26.7)	17.9 (9.8–32.7)	15.5 (8.6–28.2)

*FRF* fluid reductive factor, *LOS* length of stay in days, *MV* mechanical ventilation

*Less-than-expected = difference between observed and predicted day 1 fluids is less than − 1.5 L

^†^Expected = difference between observed and predicted day 1 fluids is between − 1.5 and 1.5 L

^‡^Greater-than-expected = difference between observed and predicted day 1 fluids is more than 1.5 L

^§^Patients may have one or more comorbidities or admission organ dysfunctions. Comorbidities selected from the Charlson-Deyo Comorbidity Index. Admission organ dysfunctions and respective definition codes adapted from Angus et al. [9]

^¶^Secondary outcomes are presented as median (IQR)

### Predicted mortality and predicted day 1 fluid resuscitation

A predicted mortality model (AUROC = 0.80) identified increasing age, comorbid neoplasm, and circulatory shock as conferring the highest risk of death (Additional file 1: Table E1). The predicted 5th to 95th percentile range of total DOF was 2.13 to 5.57 L. All patients in the inclusion cohort were predicted to receive a comorbidity-adjusted baseline DOF of 2.42 L (2.34–2.50 L). The model identified eight admission diagnoses that contributed towards a probability of receiving less fluid (fluid reductive factors, FRFs) and five admission diagnoses that contributed towards a probability of receiving more fluid compared to baseline. Patients with FRFs tended to be older than those without FRFs (66.6 vs. 54.8 years, *p* < 0.0001). As anticipated, patients with FRFs had a higher average number of organ dysfunctions present on admission than those without FRFs (2.3 vs. 2.1, *p* < 0.0001) and increased frequency of comorbid diseases (Table 1). Among the FRFs, pulmonary congestion and heart failure were associated with the highest reduction in the amount of predicted DOF resuscitation (0.43 L, 0.18–0.68 L, *p* = 0.001 and 0.42 L, 0.36–0.48 L, *p* < 0.0001, respectively). Heart failure was the most common FRF (28.5%). Acute renal failure conferred the largest increase in predicted DOF resuscitation (0.61 L, 0.55–0.67 L, *p* < 0.0001) and was present in 33.6% of patients (Additional file 1: Table E2).

Applying the predicted DOF resuscitation model, a similar number of patients who received greater-than-expected and less-than-expected DOF volumes (20.5% vs. 25.1%, respectively) were identified. Along with patients who received expected DOF volumes, no significant between-group differences in gender, age, race, or comorbid diseases were found. Patients who received greater- or and less-than-expected DOF volumes were more likely to present with a higher average number of organ dysfunctions than patients who received expected fluid volumes (2.4 and 2.5 vs. 2.0, respectively, *p* < 0.0001), particularly cardiac and renal failure (Table 1). Likewise, a greater number of organ dysfunctions were associated with incrementally increased predicted DOF (Additional file 1: Figure E1).

### Observed vs. predicted hospital mortality

**Fig. 2:**
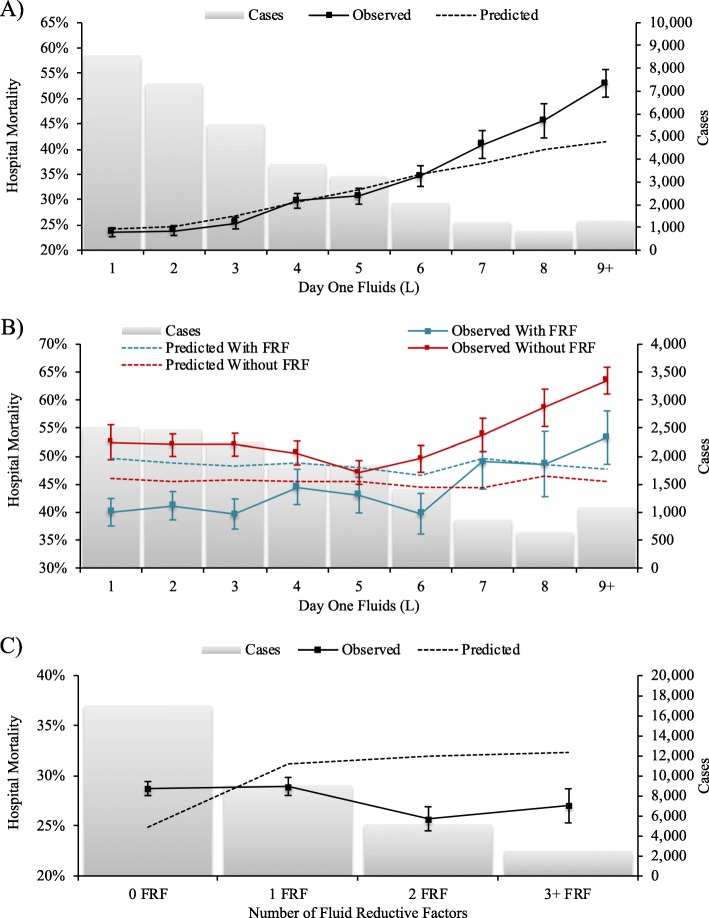
The effect of day 1 fluid resuscitation volume on hospital mortality. Observed vs. predicted hospital mortality by **a**) day 1 fluids for all patients, **b**) day 1 fluids for shock patients with or without one or more fluid reductive factors (FRFs), and **c**) number of FRFs. The difference between observed and predicted mortality is significant when 95% CI bars do not cross the line for predicted mortality

The observed hospital mortality for the inclusion cohort was 28.2%. In those where DOF exceeded 6 L, observed mortality was incrementally higher than predicted for each additional liter of DOF. For example, patients receiving 7 L of DOF experienced a significantly worse observed mortality than predicted (40.9% vs. 37.2%, *p* = 0.008) (Fig. 2a). This association was present in all patients but most notable in patients with shock. In shock patients without any FRFs, observed mortality was significantly worse than predicted at both lower and higher DOF volumes (Fig. 2b). In contrast, patients with one or more FRFs experienced better than predicted mortality at lower DOF volumes and worse than predicted mortality only after 9 or more liters of DOF. Additionally, despite higher predicted mortality, observed mortality for patients with an FRF was lower than patients without an FRF at all DOF volumes (Fig. 2b). Lastly, while predicted mortality increased as anticipated with each additional FRF, observed mortality paradoxically decreased further with the addition of a second FRF (Fig. 2c).

**Fig. 3:**
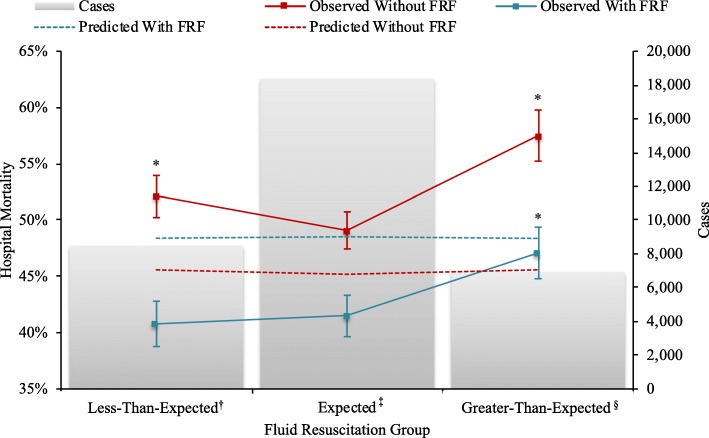
The effect of greater- or less-than-expected day 1 fluid volume resuscitation on hospital mortality. Risk-adjusted observed vs. predicted hospital mortality for patients with shock. Risk adjustment performed by adding the difference in predicted hospital mortality between expected-resuscitation and less-than-expected or greater-than-expected groups to their respective observed hospital mortality. *FRF* fluid reductive factor. Asterisk indicates statistically significant difference in observed hospital mortality when compared to the expected-resuscitation group^. †^Less-than-expected = difference between observed and predicted day 1 fluids is less than − 1.5 L. ^‡^Expected = difference between observed and predicted day 1 fluids is between − 1.5 and 1.5 L. ^§^Greater-than-expected = difference between observed and predicted day 1 fluids is more than 1.5 L

To further explore this relationship, patients were stratified by fluid resuscitation volume relative to expected using the predicted DOF model. Among all shock patients, greater-than-expected fluid resuscitation compared to expected fluid resuscitation was associated with increased risk-adjusted hospital mortality (52.3% vs. 45.0%, *p* < 0.0001). The same was true for shock patients with an FRF (47.1% vs. 41.5%, *p* < 0.0001). However, in patients with shock but without an FRF, both greater-than-expected resuscitation (57.5% vs. expected 49.2%, *p* < 0.0001) and less-than-expected resuscitation (52.1% vs. expected 49.2%, *p* = 0.037) were associated with increased risk-adjusted hospital mortality (Fig. 3 and Additional file 1: Figure E2). Regardless of relative resuscitation, the risk-adjusted observed hospital mortality was lower than predicted in shock patients with FRFs and higher than predicted in shock patients without FRFs (Fig. 3). When applied to the validation database, both the predicted DOF model (*R*^2^ = 19.1%) and predicted mortality model (AUROC 0.80, HL = 12.03, *p* = 0.15) demonstrated highly comparable performance and excellent agreement in predicted and observed outcomes irrespective of diagnosis or presence of shock (Additional file 1: Table E3 and Additional file 1: Figure E3).

Similar to all shock patients, the observed mortality for patients with septic shock was incrementally greater than predicted mortality with incrementally great DOF volumes, achieving statistical significance at 7 L of DOF (36.9% vs. 32.9%, *p* = 0.019) (Additional file 1: Figure E4). Risk-adjusted hospital mortality was comparatively worse in septic shock patients who received greater-than-expected DOF volumes both with (40.4% vs. expected 34.7%, *p* = 0.001) and without (51.5% vs. expected 42.1%, *p* < 0.0001) an FRF (Additional file 1: Figure E5).

### Secondary outcomes

**Fig. 4:**
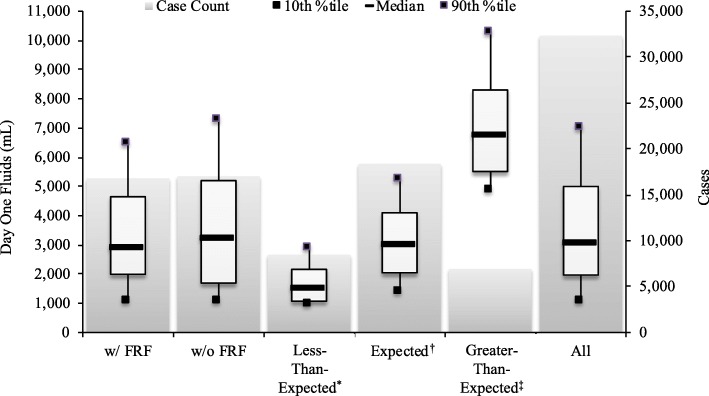
Variability in day 1 fluid resuscitation volumes. Modified box-and-whiskers plots (box ends indicate 25th and 75th percentiles, whiskers indicate 10th and 90th percentiles, and middle lines indicate medians) for prescribed day 1 fluids. Differences in medians between w/ FRF and w/o FRF groups, as well as between less-than-expected, expected, and greater-than-expected resuscitation groups, are statistically significant (*p* < 0.0001). *FRF* fluid reductive factor. *Less-than-expected = difference between observed and predicted day 1 fluids is less than − 1.5 L. ^†^Expected = difference between observed and predicted day 1 fluids is between − 1.5 and 1.5 L. ^‡^Greater-than-expected = difference between observed and predicted day 1 fluids is more than 1.5 L

For the entire inclusion cohort, there was a wide range of prescribed DOF resuscitation volumes (3.0 L, IQR 1.1 L–5.0 L). DOF volume was slightly higher among patients with an FRF (3.2 L, IQR 1.7 L–5.2 L) compared to patients without an FRF (2.9 L, IQR2.0 L–4.7 L, *p* < 0.0001). Additionally, there were marked differences in DOF between expected (3.0 L, IQR 2.1 L–4.1 L), greater-than-expected (6.8 L, IQR 5.5 L–8.3 L), and less-than-expected (1.5 L, IQR 1.1 L–2.2 L, *p* < 0.0001 for all comparisons) resuscitation groups (Fig. 4).

Median hospital LOS was 6 days (IQR 2–10 days). Median ICU LOS was 3 days (IQR 2–6 days), and the median duration of mechanical ventilation was 3 days (IQR 2–6 days). Median cost for the admission was $15,516 (IQR $8638–$28,185). Compared to patients without an FRF, patients with an FRF had higher median hospital LOS (7 vs. 4 days, *p* < 0.0001), ICU LOS (4 vs. 2 days, *p* < 0.0001), duration of mechanical ventilation (3 vs. 2 days, *p* < 0.0001), and cost ($18,504 vs. $12,724, *p* < 0.0001) (Table 1).

### Institutional variability in fluid resuscitation practices

When assessed by the presence or absence of an FRF and by relative DOF resuscitation, there were no significant differences in observed DOF volumes by hospital size, teaching status, location, or geographic region. The difference between observed and predicted fluid resuscitation was also preserved across hospital demographics (Additional file 1: Table E4).

## Discussion

In this retrospective outcome analysis using the 2013 Premier and 2013–2016 Truven databases, we found highly variable day 1 fluid resuscitation practices among mechanically ventilated patients with hemodynamic shock from diverse etiologies. Our results are consistent with prior observations associating over-resuscitation with adverse outcomes. However, we also found that lower-than-expected day 1 fluid resuscitation, while beneficial among patients with certain diagnoses predictive of receiving less fluids (e.g., heart failure, end-stage renal disease), was associated with increased risk-adjusted hospital mortality among a relatively healthier patient cohort without such diagnoses; a U-shaped relationship.

Initial fluid resuscitation practices for patients with hemodynamic shock remain controversial. Recently published data from a prospective randomized control pilot study of patients with severe sepsis and septic shock demonstrated no increase in adverse outcomes using a restrictive fluid resuscitation strategy in the first 72 h of care [11]. Larger multicenter trials comparing fluid-restrictive and fluid-liberal resuscitation are currently underway [12]. Nevertheless, the non-uniform response to fluid resuscitation between patients with and without an FRF observed in our study suggests widely inclusive fluid resuscitation strategies in usual care and outside of study protocols may not be appropriate for all patients. Moreover, while over-resuscitation is increasingly recognized as harmful to patients with shock [2–8] and fluid-restrictive strategies are currently being evaluated in large multicenter trials, our findings stress that under-resuscitation may also be harmful particularly for a subgroup of patients without FRFs.

Only half of shock patients respond positively to a volume challenge, indicating that many may have effective circulating fluid volumes despite macrocirculatory shock [13]. Diagnoses identified as FRFs frequently include volume overload as a primary or secondary feature, potentially explaining the observed mortality benefit of receiving less-than-expected fluid volumes among patients with at least one of these conditions. Patients without an FRF were generally younger and had fewer comorbidities and acute organ dysfunctions present on admission. They also had lower predicted hospital mortality compared to patients with an FRF. Nonetheless, hospital mortality for patients without an FRF was increased with higher and lower fluid volumes and with greater- and less-than-expected fluid resuscitation. These findings challenge the assumption that younger and presumably healthier patients are more tolerant of a wide range of fluid resuscitation volumes. Likewise, we observed significantly lower ICU and hospital resource utilization and lower hospital costs among patients without an FRF despite higher-than-predicted risk-adjusted hospital mortality. This incongruity may reflect relatively less attentive care-titration for a population presumed to have lower risk for further clinical decompensation.

To our knowledge, this study represents the largest novel analysis of fluid resuscitation outcomes for a wide spectrum of shock states. We specifically focused on patients requiring mechanical ventilation as they have the highest comorbidity burden and risk for hospital mortality. To adjust for confounding by disease severity, we developed predicted mortality and predicted DOF models. By uniquely evaluating the impact of greater- or less-than-expected fluid resuscitation, we expand on previous studies limited to comparisons of absolute fluid volumes in septic shock alone [8, 14]. Furthermore, we validated our predictive models and findings in a second large database that includes patients discharged as recently as 2016 and, pertaining to septic shock, subsequent to the ProCESS [15], ARISE [16], and ProMISe [17] trials.

A uniform limitation of many administrative database analyses is the lack of patient-level severity of illness scoring. However, comorbidity-based risk-adjustment indices (e.g., Charlson index AUROC = 0.67) have been shown to perform similarly well as severity of illness scores (APACHE II AUROC = 0.805 and SAPS II AUROC = 0.843) in predicting short-term mortality following ICU admission [18–20]. The discriminative ability of our comorbidity-based mortality model, while still dependent on ICD-9-CM-based coding instead of physiologic variables, compares favorably (AUROC = 0.80). In addition, the linear relationship between prescribed fluid volumes and predicted mortality observed in our model is consistent with prior observational studies suggesting a positive correlation between administered fluids and mortality. The range of predicted DOF accurately correlated with the range of observed DOF, and the ± 1.5-L DOF cutoff was validated by the continuous deviation of observed from predicted mortality rates both above and below this critical value. Nonetheless, we cannot exclude the possibility that variance with respect to predicted versus observed DOF may be related to the performance of our model. Thus, while acknowledging the limitation of the current administrative dataset analyses, future prospective studies should evaluate the temporal relationship between prescribed fluid volumes and mechanical ventilation or other physiologic variables. Such an approach would facilitate further categorization of disease severity, for example by computing Sequential Organ Assessment (SOFA) scores for patients with sepsis, and elucidate the causal relationship between suboptimal fluid resuscitation and adverse outcomes.

Additional potential for confounding arises in administrative database analyses from systematic underreporting by individual hospitals and absent documentation for specific patients. To mitigate this potential bias, we excluded hospitals where the 25th percentile for recorded DOF was below 250 mL and any cases where the DOF was less than 1 L (185 hospitals, 33%). The potential for ascertainment bias, however, exists as a result of excluding patients who may be admitted late in the calendar hospital day and hospitals with systematic underreporting of administered fluid volumes. With respect to identifying patients with shock, we attempted to address this by defining shock according to whether the subject was prescribed a vasopressor on hospital day 1 or 2. Furthermore, while isolating a higher risk population, patients with shock who require mechanical ventilation may represent a phenotypically distinct but heterogeneous subgroup. In separate analyses, we observed comparable associations in acutely resuscitated patients regardless of the need for ventilator support (unpublished data). Categorization of different shock states in administrative data is limited without access to patient-specific physiologic variables. For the purposes of the current analysis, we refer to all shock states as “undifferentiated shock” while acknowledging that bedside clinical assessment is likely to have informed a more precise etiology of shock that contributed to fluid resuscitation decisions. Finally, the present analysis does not distinguish between types of fluid administered that is the focus of ongoing studies. Though resuscitation with crystalloid rather than colloid fluids may have contributed to variability in DOF volumes [21], fluid type has not been consistently associated with differences in shock resuscitation outcomes [21–23].

Our study focused on early, guideline-informed DOF administration in shock. Future analyses are planned to evaluate DOF volumes for patients with delayed versus direct ICU admission, hospitals where patients receive very small volumes of DOF, the impact of specific shock states, as well as outcomes related to fluid administration beyond day 1 through hospital discharge. Further validation analyses in separate cohorts will be required to confirm the performance our novel risk-adjusted models. Institutional differences, including hospital size, teaching status, location, or geographic region, were not associated with variability in DOF resuscitation. While likely not a function exclusively of organizational or hospital-level factors, these data do not preclude that there may be significant individual- or team-level practice variability. Clinician-level variability in resuscitation decisions could also enhance future analyses of treatment variations. Taken together, these findings compellingly point to the importance of ongoing prospective clinical trials of physiologically informed, patient-centered fluid resuscitation for shock using dynamic measures of circulatory effectiveness rather than uniformly empiric strategies.

## Conclusions

Among mechanically ventilated patients with diverse etiologies of shock, this large retrospective analysis highlights extensive treatment variability including frequent greater- or less-than-expected fluid volume resuscitation. There was a non-uniform association with risk-adjusted hospital mortality, which was surprisingly worse in patients without a fluid reductive factor. These findings support further evaluation of which patients may potentially benefit or be harmed by restrictive and liberal fluid resuscitation strategies in usual care.

## Supplementary information

**Additional file 1.** Supplementary_Materials. Additional analyses and figures

## Data Availability

The data that support the findings of this study are available from Premier Hospital Database (Premier Inc., Charlotte, NC, USA) and MarketScan Hospital Drug Database (Truven Health Analytics Inc., Ann Arbor, MI, USA) but restrictions apply to the availability of these data, which were used under license for the current study, and so are not publicly available. Data are however available from the authors upon reasonable request and with permission of Premier, Inc. and Truven Inc.

## Abbreviations

APACHE II: Acute Physiology and Chronic Health Evaluation II
AUROC: Area under receiver operating characteristic
DOF: Day 1 fluid
FRF: Fluid reductive factor
ICU: Intensive care unit
LOS: Length of stay
SAPS II: Simplified Acute Physiology Score II

## References

[1] Bikdeli B, Strait KM, Dharmarajan K, Li SX, Mody P, Partovian C (2015). Intravenous fluids in acute decompensated heart failure. JACC Heart Fail.

[2] Lee J, de Louw E, Niemi M, Nelson R, Mark RG, Celi LA (2015). Association between fluid balance and survival in critically ill patients. J Intern Med.

[3] Boyd JH, Forbes J, Nakada TA, Walley KR, Russell JA (2011). Fluid resuscitation in septic shock: a positive fluid balance and elevated central venous pressure are associated with increased mortality. Crit Care Med.

[4] Malbrain ML, Marik PE, Witters I, Cordemans C, Kirkpatrick AW, Roberts DJ (2014). Fluid overload, de-resuscitation, and outcomes in critically ill or injured patients: a systematic review with suggestions for clinical practice. Anaesthesiol Intensive Ther.

[5] Kelm DJ, Perrin JT, Cartin-Ceba R, Gajic O, Schenck L, Kennedy CC (2015). Fluid overload in patients with severe sepsis and septic shock treated with early goal-directed therapy is associated with increased acute need for fluid-related medical interventions and hospital death. Shock..

[6] Claure-Del Granado R, Mehta RL (2016). Fluid overload in the ICU: evaluation and management. BMC Nephrol.

[7] Acheampong A, Vincent JL (2015). A positive fluid balance is an independent prognostic factor in patients with sepsis. Crit Care.

[8] Marik PE, Linde-Zwirble WT, Bittner EA, Sahatjian J, Hansell D (2017). Fluid administration in severe sepsis and septic shock, patterns and outcomes: an analysis of a large national database. Intensive Care Med.

[9] Angus DC, Linde-Zwirble WT, Lidicker J, Clermont G, Carcillo J, Pinsky MR (2001). Epidemiology of severe sepsis in the United States: analysis of incidence, outcome, and associated costs of care. Crit Care Med.

[10] Deyo RA, Cherkin DC, Ciol MA (1992). Adapting a clinical comorbidity index for use with ICD-9-CM administrative databases. J Clin Epidemiol.

[11] Corl KA, Prodromou M, Merchant RC, Gareen I, Marks S, Banerjee D (2019). The restrictive IV fluid trial in severe sepsis and septic shock (RIFTS): a randomized pilot study. Crit Care Med.

[12] Self WH, Semler MW, Bellomo R, Brown SM, BP dB, Exline MC (2018). Liberal versus restrictive intravenous fluid therapy for early septic shock: rationale for a randomized trial. Ann Emerg Med.

[13] Michard F, Teboul JL (2002). Predicting fluid responsiveness in ICU patients: a critical analysis of the evidence. Chest..

[14] de Oliveira FS, Freitas FG, Ferreira EM, de Castro I, Bafi AT, de Azevedo LC (2015). Positive fluid balance as a prognostic factor for mortality and acute kidney injury in severe sepsis and septic shock. J Crit Care.

[15] Yealy DM, Kellum JA, Huang DT, Barnato AE, Weissfeld LA, Pike F (2014). A randomized trial of protocol-based care for early septic shock. N Engl J Med.

[16] Peake SL, Delaney A, Bailey M, Bellomo R, Cameron PA, Cooper DJ (2014). Goal-directed resuscitation for patients with early septic shock. N Engl J Med.

[17] Mouncey PR, Osborn TM, Power GS, Harrison DA, Sadique MZ, Grieve RD (2015). Trial of early, goal-directed resuscitation for septic shock. N Engl J Med.

[18] Yang M, Mehta HB, Bali V, Gupta P, Wang X, Johnson ML (2015). Which risk-adjustment index performs better in predicting 30-day mortality? A systematic review and meta-analysis. J Eval Clin Pract.

[19] Livingston BM, MacKirdy FN, Howie JC, Jones R, Norrie JD (2000). Assessment of the performance of five intensive care scoring models within a large Scottish database. Crit Care Med.

[20] Poses RM, McClish DK, Smith WR, Bekes C, Scott WE (1996). Prediction of survival of critically ill patients by admission comorbidity. J Clin Epidemiol.

[21] Finfer S, Bellomo R, Boyce N, French J, Myburgh J, Norton R (2004). A comparison of albumin and saline for fluid resuscitation in the intensive care unit. N Engl J Med.

[22] Young P, Bailey M, Beasley R, Henderson S, Mackle D, McArthur C (2015). Effect of a buffered crystalloid solution vs saline on acute kidney injury among patients in the intensive care unit: the SPLIT randomized clinical trial. JAMA..

[23] Caironi P, Tognoni G, Masson S, Fumagalli R, Pesenti A, Romero M (2014). Albumin replacement in patients with severe sepsis or septic shock. N Engl J Med.

