# Supplementary material for: Variability in usual care fluid resuscitation and risk-adjusted outcomes for mechanically ventilated patients in shock
Source: Crit Care. 2020 Jan 28;24:25. doi: 10.1186/s13054-020-2734-9 (PMC6986034; doi:10.1186/s13054-020-2734-9)
Supplement: Supplementary file 1 — Additional file 1. Supplementary_Materials. Additional analyses and figures [file 13054_2020_2734_MOESM1_ESM.pdf]

Retrospective Analysis of Usual Care Fluid Resuscitation and Risk-Adjusted Outcomes for Mechanically Ventilated Patients in Shock

Jason N. Mansoori, MD, Walter Linde-Zwirble, Peter C. Hou, MD, Edward P. Havranek, MD, Ivor S. Douglas, MD

Online Supplement

Data Source 1

Case Mix Adjustment 1

Statistical Analysis 1

Model Development 1

Supplementary Material References 2

Supplementary Material Tables 3

Table E1. Age groups and admission diagnoses associated with risk of death 3

Table E2. Factors associated with prdicted day one fluid resuscitation 4

Table E3. Day one fluid volume resuscitation by hospital demographic factors 5

Table E4. Day one fluid volume resuscitation by hospital demographic factors 6

Supplementary Material Figures 7

Figure E1. Distribution of predicted day one fluids for all included patients by acute organ dysfunctions present on admission 7

Figure E2 The effect of differences between observed and predicted day one fluid resuscitation and presence of an FRF on hospital mortality in patients with shock 8

Figure E3. Similar effects of day one fluid resuscitation volume on hospital mortality in the validation database 9

Figure E4. The effect of day one fluid resuscitation volume and presence of an FRF on hospital mortality in patients with septic shock 10

Figure E5. The effect of day one fluid volume under or over-resuscitation in patients with septic shock 11

Data Sources

The Premier Hospital Database is an electronic, administrative-level data repository of patients discharged in the USA, representing approximately 40% of patients discharged nationally including more than 5 million patient visits per year (1). The database contains International Classification of Diseases, Ninth Revision, Clinical Modification (ICD-9-CM) diagnoses including present on admission information and ICD-9-CM procedures by date. Information is collected on all patients within all hospital treatment areas, additionally including patient demographic data and a date stamped log with quantity, cost, and charge details for all invoiced items, such as medications, laboratory orders, diagnostic and therapeutic services. Participating hospitals are geographically diverse, primarily non-profit and non-governmental, and include community and teaching hospitals from rural and urban areas (1). Fewer than one percent of patient records have missing data for most components, while fewer than 0.01 percent have missing data for key components, such as demographics and diagnostics information (2). Currently, more than 375 studies utilizing the Premier Hospital Database have been published in peer-reviewed journals across multiple disciplines (1). The Truven Health MarketScan Hospital Drug Database contains clinical data on inpatient episodes for all patients discharged from approximately 600 United States Hospitals. These records include admission and discharge dates, patient demographics and provider specialty, and specific codes for diagnosis, procedures, drug administration, and facility descriptors. This database has been shown to be representative of acute care hospitals in the United States (3-5).

Case Mix Adjustment

We constructed binary response models for hospital mortality and a linear analysis of variance model for fluids on day one, using patient age and codes for acute conditions present on admission including acute organ dysfunctions, anemia, electrolyte disorders and altered awareness (280.0, 281.0, 282.0-.3, 287.7, 283, 284.9, 285.0, 285.1, 285.2, 285.8, 276, 780.0), and a number of chronic conditions: heart failure (398.91, 402.91, 425.4-.9, 428); chronic renal failure; end stage renal disease; neoplasm of digestive, bone, genito-urinary and lymphoma (150-159, 170-171, 179-189, 200-208); pulmonary and central nervous system neoplasm (160-165, 191-192); metastatic neoplasm (196-199); dysrhythmias and conduction disorders (426.0-0.1, 427.3-.4, 785.0, 996.01, 996.04); hypertensive kidney disease (403.01, 403.11, 403.91, 404.01-.03, 404.11-.13, 404.91-.93); post-inflammatory pulmonary fibrosis (515); pulmonary heart disease (416); pulmonary collapse (518.0); hypertension with complications (401.0, 402-05); and complex liver disease (456.0, 570, 571.1, 572.2-.8).

Statistical Analysis

Predicted hospital mortality model fit was assessed using likelihood r-square, chi-square dispersion, area under the ROC curve, and the Hosmer-Lemeshow C statistic. The predicted DOF resuscitation model was assessed using sum of squares, R-squared, and F-ratios. Continuous data were compared by Mann-Whitney U test and categorical data by Chi-square or Fisher's exact test as appropriate. The databases were constructed in FoxPro (Microsoft Corp., Redmond WA, USA) and analyses were conducted in Data Desk (Data Description, Ithaca NY, USA).

Model Development

The initial models were developed using a systematic review process looking at all recorded patient factors. The initial development set from the Premier database was split into two random sets of hospitals. An iterative review of a series of models looked at the residuals for each condition as factors were added and removed from the model. This process examined all possible factors that were present in at least 2.5% of the population and included those that were at least as large a factor as the difference between being 40 and 50 years of age. The same set of factors was then identified in the control set of the other half of the hospitals. The model coefficients from the two models were within the statistical uncertainty for each factor. Because of the size of the dataset and the small number of predictors, there was no significant risk of over fitting. This same set of model predictors was then applied to the Truven data to properly calibrate for differences that may occur overall from a different set of hospitals and different years of care.

Predicted Mortality

Our predicted mortality model identified 11 admission diagnoses that were statistically significantly associated with increased mortality, including five that overlapped with covariates within the predicted DOF model (Supplementary Table E1). The model accounted for 65.61% of variation and also demonstrated good calibration and performance (HL=14.81, p=0.063, AUROC=0.80). Risk of death increased with age, and the oldest age group (75-99 years of age) also had the highest observed frequency (24.5%). Of the admission diagnoses, the neoplastic diseases, including pulmonary and CNS neoplasm followed by metastatic neoplasm, conferred

the highest risk of death. Of the conditions comprising each segment, a circulatory diagnosis with shock conferred the highest risk of death. This was followed by other (non-infectious) diagnoses with shock and septic shock. Conditions without shock, as anticipated, conferred lower risks of death (Supplementary Table E1).

Predicted Day One Fluid Resuscitation

The predicted day one fluid resuscitation model accounted for 18.9% (R^2) of variation. Septic shock patients were predicted to receive the most additional fluid (2.15L, 2.11-2.19L, $p < 0.0001$). With the exception of cardiac (47.4% vs. 41.1%, $p<0.0001$) and renal organ dysfunctions (38.8% vs. 28.5%, $p<0.0001$), other organ dysfunctions were similar between patients with and without an FRF. With the exception of pulmonary congestion and pulmonary collapse, the remaining six FRFs were codes for chronic conditions. The fluid additive factors consisted largely of codes for acute organ failures and electrolyte disorders (Supplementary Table E2).

References

1. Premier Life Sciences Research and Analytics Division. Premier healthcare database: data that informs and performs. 2017 [cited 2017 March 1]. Available from: https://learn.premierinc.com/pharmacy-and-research/premier-healthcare-database-whitepaper.
2. Fisher BT, Lindenauer PK, Feudtner C. In-Hospital Databases. Pharmacoepidemiology: Wiley-Blackwell; 2012. p. 244-258.
3. Baggs J, Fridkin SK, Pollack LA, Srinivasan A, Jernigan JA. Estimating National Trends in Inpatient Antibiotic Use Among US Hospitals From 2006 to 2012. *JAMA Intern Med* 2016; 176: 1639-1648.
4. Baggs J, Jernigan JA, Halpin AL, Epstein L, Hatfield KM, McDonald LC. Risk of Subsequent Sepsis Within 90 Days After a Hospital Stay by Type of Antibiotic Exposure. *Clin Infect Dis* 2018; 66: 1004-1012.
5. Dellit TH, Owens RC, McGowan JE, Jr., Gerding DN, Weinstein RA, Burke JP, Huskins WC, Paterson DL, Fishman NO, Carpenter CF, Brennan PJ, Billeter M, Hooton TM, Infectious Diseases Society of A, Society for Healthcare Epidemiology of A. Infectious Diseases Society of America and the Society for Healthcare Epidemiology of America guidelines for developing an institutional program to enhance antimicrobial stewardship. *Clin Infect Dis* 2007; 44: 159-177.
6. Angus DC, Linde-Zwirble WT, Lidicker J, Clermont G, Carcillo J, Pinsky MR. Epidemiology of severe sepsis in the United States: analysis of incidence, outcome, and associated costs of care. *Crit Care Med* 2001; 29: 1303-1310.

Supplementary Tables

	Frequency, %	Scaled Score	p-value
**Age Group (years)**			
18-24	3.8	0	<0.0001
25-34	6.4	2	<0.0001
35-44	7.8	2	<0.0001
45-54	15.3	3	<0.0001
55-64	21.7	5	0.671
65-74	20.5	8	<0.0001
75-99	24.5	15	<0.0001
**Admission Diagnoses**			
Acute Hematologic Failure*[,†]	11.9	5	<0.0001
Acute CNS Failure[†]	25.1	7	<0.0001
Acute Renal Failure[†]	33.6	2	<0.0001
Acute Hepatic Failure[†]	4.7	6	<0.0001
Altered Awareness	7.4	8	<0.0001
Neoplasm of Digestive, Bone, GU, Lymphoma	3.6	10	<0.0001
Dysrhythmias and Conduction Disorders	22.8	3	<0.0001
Metastatic Neoplasm	3.0	12	<0.0001
Pulmonary and CNS Neoplasm	2.4	13	<0.0001
Hypertensive Kidney Disease[†]	5.6	3	0.002
Complex Liver Disease	8.0	8	<0.0001
**Segments**			
Circulatory Diagnosis With Shock	12.3	36	<0.0001
Circulatory Diagnosis Without Shock	8.8	14	0.009
Infection Diagnosis With Shock	26.7	19	<0.0001
Infection Diagnosis Without Shock	30.6	1	<0.0001
Other Diagnosis With Shock	5.3	25	<0.0001
Other Diagnosis Without Shock	16.4	0	<0.0001

Table E1. Age groups and admission diagnoses associated with risk of death.
* Represents acute venous or arterial thromboembolism
[†] Admission diagnoses also included in predicted day one fluid model

	Frequency, %	Liter Change	p-value
**Male Gender**	52.6	0.08	<0.0001
**Age Group (years)**			<0.0001
18-24	3.8	0.68	<0.0001
25-34	6.4	0.56	<0.0001
35-44	7.8	0.56	<0.0001
45-54	15.3	0.40	0.623
55-64	21.7	0.30	0.002
65-74	20.5	0.20	<0.0001
75-99	24.5	0.00	<0.0001
**Admission Diagnoses**			
**Fluid Additive Factors**			
Acute Hematologic Failure*	11.9	0.54	<0.0001
Acute CNS Failure	25.1	0.07	0.016
Acute Renal Failure	33.6	0.61	<0.0001
Acute Hepatic Failure	4.7	0.45	<0.0001
Electrolyte Disorders	55.2	0.38	<0.0001
**Fluid Reductive Factors**			
End Stage Renal Disease	5.2	-0.23	<0.0001
Pulmonary Congestion	0.9	-0.43	0.001
Heart Failure	28.5	-0.42	<0.0001
Chronic Bronchitis	13.2	-0.37	<0.0001
Post-inflammatory Pulmonary Fibrosis	1.4	-0.28	0.006
Pulmonary Heart Disease	7.0	-0.15	0.002
Hypertensive Kidney Disease	20.7	-0.29	<0.0001
Pulmonary Collapse	4.8	-0.22	<0.0001
**Segments**			
Circulatory Diagnosis With Shock	12.3	1.61	<0.0001
Circulatory Diagnosis Without Shock	8.8	0.32	<0.0001
Infection Diagnosis With Shock	26.7	2.15	<0.0001
Infection Diagnosis Without Shock	30.6	0.66	<0.0001
Other Diagnosis With Shock	5.3	1.02	0.203
Other Diagnosis Without Shock	16.4	0.00	<0.0001

Table E2. Factors associated with predicted day one fluid resuscitation. Model predicted a baseline of 2.42L day one fluid resuscitation for all patients.

*Represents acute venous or arterial thromboembolism

		Observed Day-One Fluid	Predicted Day-One Fluid	Observed Mortality	Predicted Mortality
**Diagnosis**	**Count, n (%)**	**Mean [Median] (L)**	**Mean [Median] (L)**	**Mean**	**Mean**
Circulatory With Shock	5,020 (14.5)	4.4 [3.5]	4.7 [4.7]	61.3%	62.3%
Circulatory Without Shock	3,164 (9.2)	2.8 [2.1]	3.1 [3.0]	34.2%	32.9%
Infection With Shock	8,846 (25.6)	5.1 [4.4]	5.3 [5.2]	40.0%	40.9%
Infection Without Shock	9,730 (28.2)	3.4 [2.8]	3.5 [3.5]	10.4%	11.1%
Other With Shock	2,207 (6.4)	4.2 [3.4]	4.1 [4.1]	42.2%	45.2%
Other Without No Shock	5,573 (16.1)	2.8 [2.1]	3.0 [3.1]	7.4%	7.2%
All	34,540 (100)	3.9 [3.0]	4.1 [3.9]	29.1%	29.7%

Table E3. Agreement between observed and predicted day-one fluid and hospital mortality. Observed and predicted data are from the 2013-2016 MarketScan Hospital Drug Database (Truven Health Analytics Inc., Ann Arbor, MI, USA) applying predictive models developed in the 2013 Premier Hospital Database (Premier, Inc.).

	w/ FRF	w/o FRF	Less-Than-Expected*	Expected[†]	Greater-Than-Expected[‡]	All
**Hospital Size**						
<200 Beds	3.5 [3.5]	3.9 [3.9]	1.7 [4.0]	3.3 [3.4]	7.2 [4.0]	3.7 [3.7]
200-399 Beds	3.4 [3.5]	4.0 [3.9]	1.7 [4.1]	3.2 [3.4]	7.3 [3.9]	3.7 [3.7]
400+ Beds	3.4 [3.4]	3.8 [3.8]	1.7 [4.0]	3.2 [3.4]	7.2 [3.8]	3.6 [3.6]
**Teaching Status**						
Non-Teaching	3.4 [3.5]	3.9 [3.9]	1.7 [4.1]	3.2 [3.4]	7.2 [3.9]	3.6 [3.7]
Teaching	3.6 [3.5]	3.9 [3.8]	1.7 [4.0]	3.2 [3.4]	7.3 [3.8]	3.7 [3.6]
**Location**[§]						
Rural	3.3 [3.4]	3.6 [3.8]	1.7 [4.0]	3.1 [3.2]	7.0 [3.8]	3.4 [3.6]
Urban	3.5 [3.5]	3.9 [3.9]	1.7 [4.0]	3.2 [3.4]	7.3 [3.9]	3.7 [3.7]
**Region**[§]						
Midwest	3.4 [3.4]	3.9 [3.9]	1.7 [4.0]	3.2 [3.4]	7.0 [3.8]	3.6 [3.6]
Northeast	3.4 [3.4]	3.6 [3.7]	1.6 [3.9]	3.1 [3.3]	7.0 [3.7]	3.5 [3.6]
South	3.4 [3.5]	3.9 [4.0]	1.7 [4.1]	3.2 [3.4]	7.2 [3.9]	3.6 [3.7]
West	3.8 [3.6]	4.0 [3.9]	1.8 [4.1]	3.3 [3.5]	7.7 [3.9]	3.9 [3.8]

Table E4. Day one fluid volume resuscitation by hospital demographic factors. Observed [Predicted] day one fluid volume resuscitation in liters.

FRF = Fluid Reductive Factor

* Less-Than-Expected = difference between observed and predicted day one fluids is less than -1.5L

[†] Expected = difference between observed and predicted day one fluids is between -1.5L and 1.5L

[‡] Greater-Than-Expected = difference between observed and predicted day one fluids is more than 1.5L

[§] Location and geographic region defined as per coding within the Premier Hospital Database

Supplementary Figures

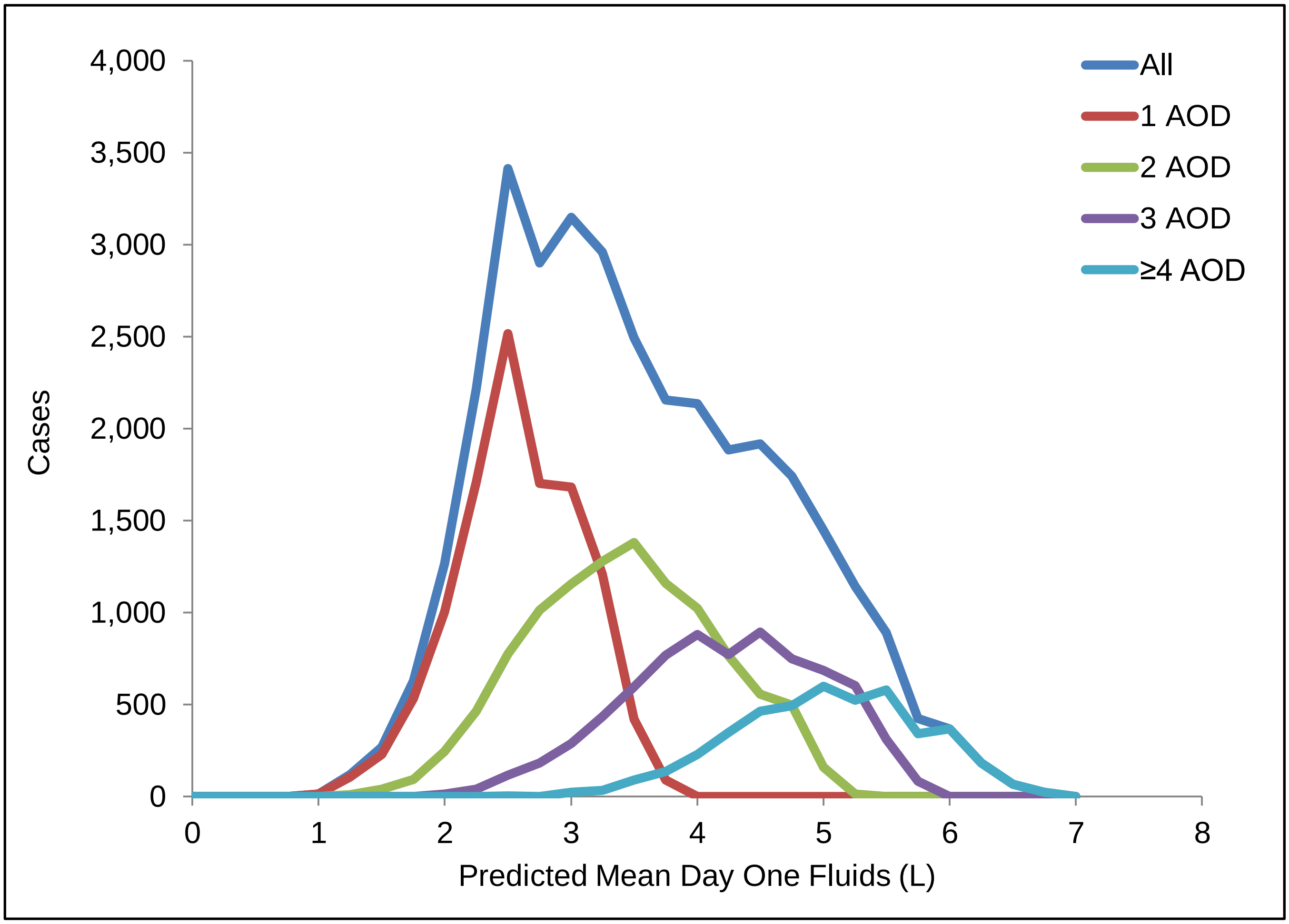

Figure E1. Distribution of predicted day one fluids for all included patients by acute organ dysfunctions present on admission. Admission organ failures and respective definition codes adapted from Angus et al., 2001 (6).
AOD = Acute Organ Dysfunction

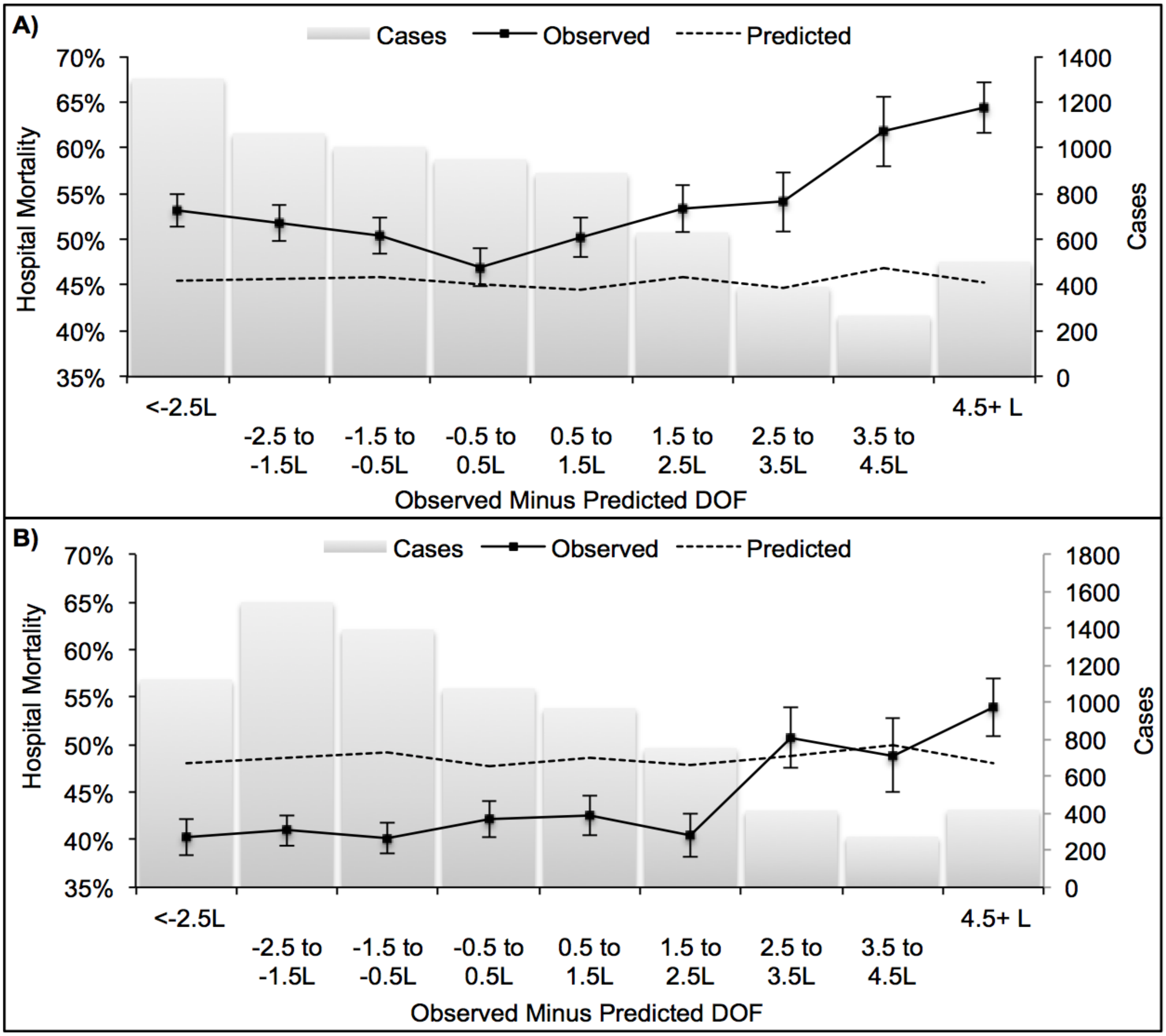

Figure E2. The effect of differences between observed and predicted day one fluid resuscitation and presence of an FRF on hospital mortality in patients with shock. Observed vs. predicted hospital mortality in patients with shock by A) observed minus predicted DOF in patients without an FRF and B) with an FRF. The difference between observed and predicted mortality is significant when 95% CI bars do not cross the line for predicted mortality.
DOF = Day One Fluid

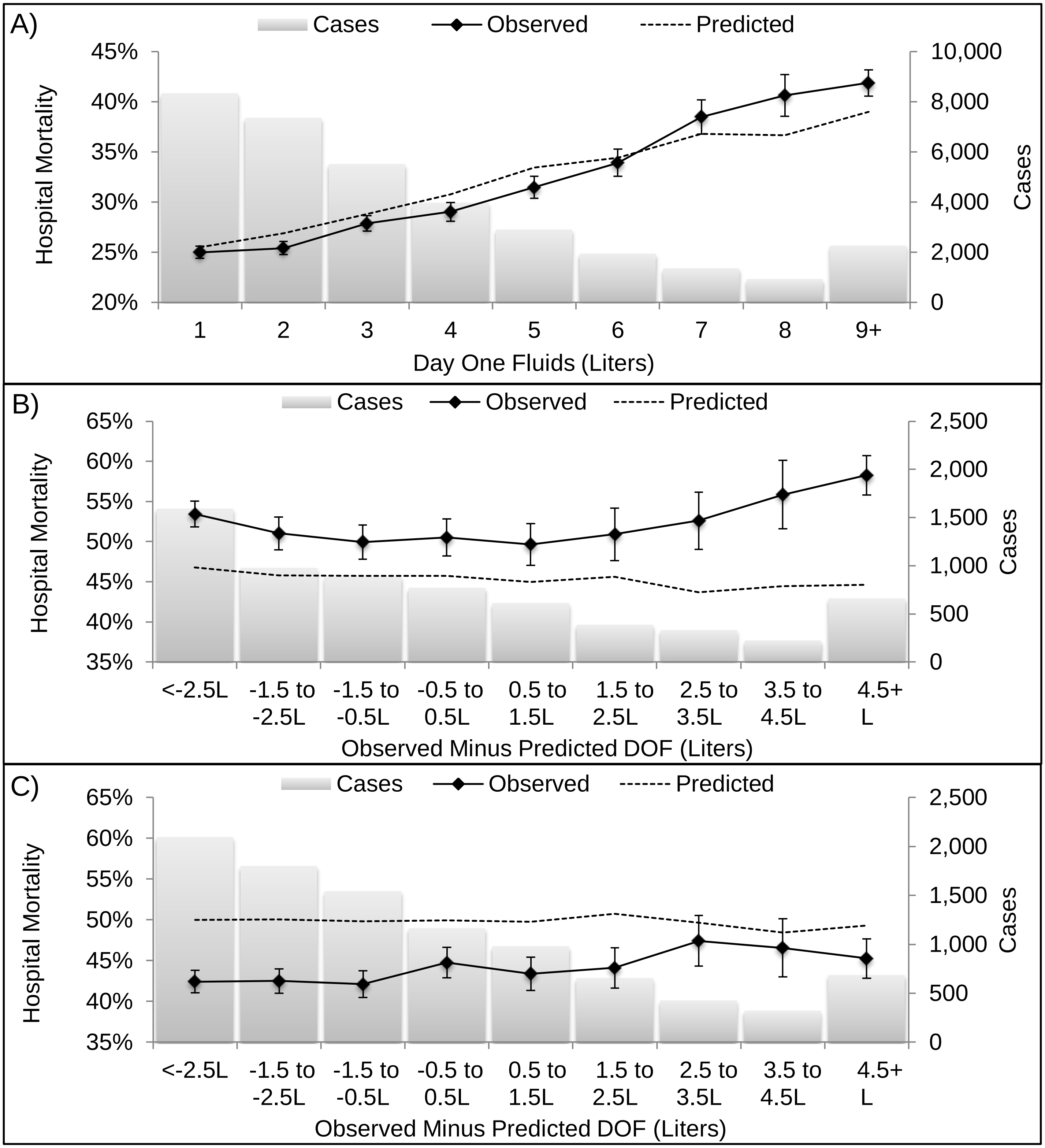

Figure E3. Similar effects of day one fluid resuscitation volume on hospital mortality in the validation database. Observed vs. predicted hospital mortality by A) day one fluids for all patients, B) difference in observed and predicted day one fluid for shock patients without one or more fluid reductive factors (FRFs), and C) with one or more FRFs. The difference between observed and predicted mortality is significant when 95 percent CI bars do not cross the line for predicted mortality.
DOF = Day One Fluid

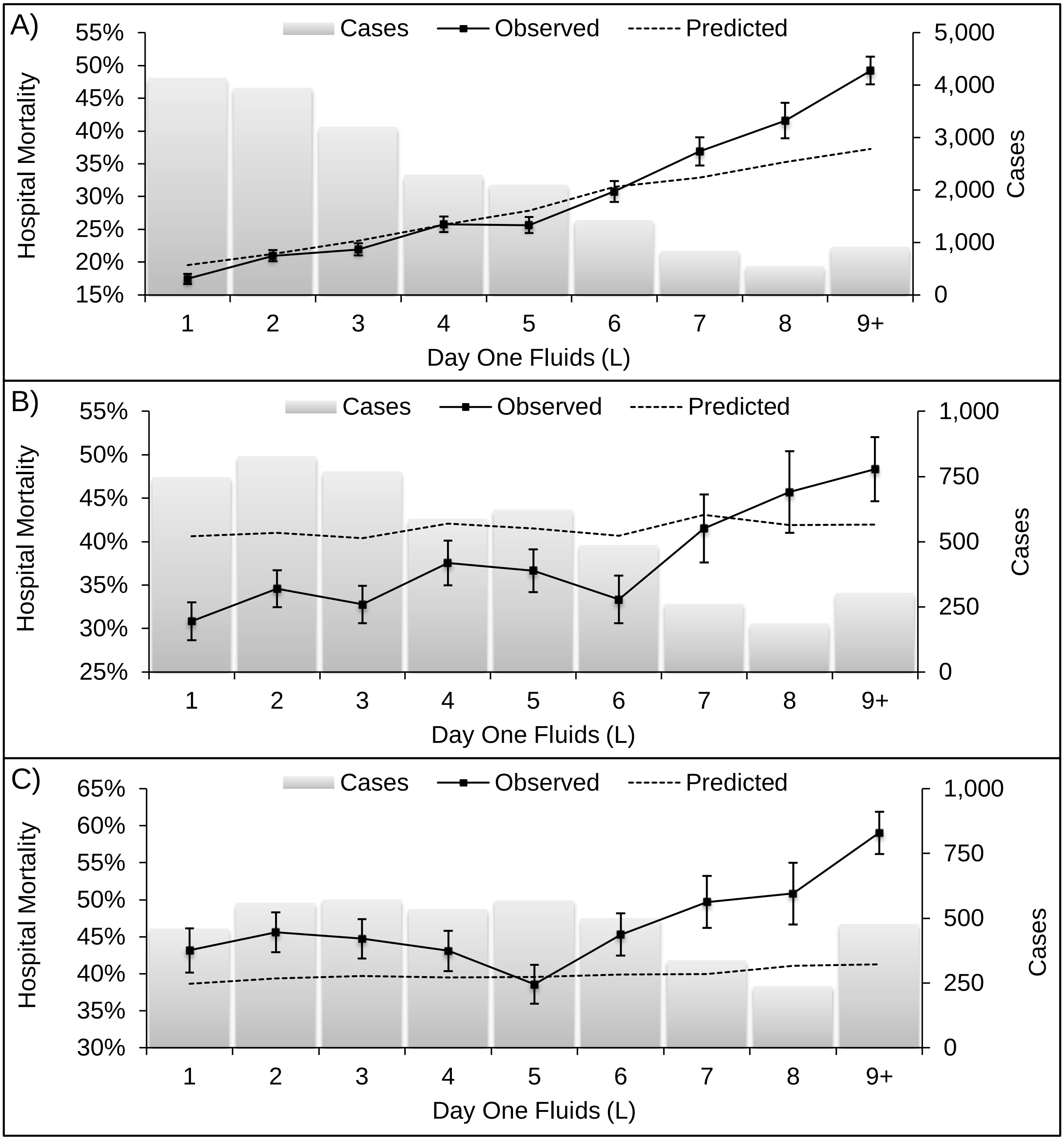

Figure E4. The effect of day one fluid resuscitation volume on hospital mortality in patients with septic shock. Observed vs. predicted hospital mortality in patients with septic shock by A) day one fluids, B) day one fluids in patients with one or more fluid reductive factor (FRF), and C) day one fluids in patients without an FRF. The difference between observed and predicted mortality is significant when 95% CI bars do not cross the line for predicted mortality.
DOF = Day One Fluid

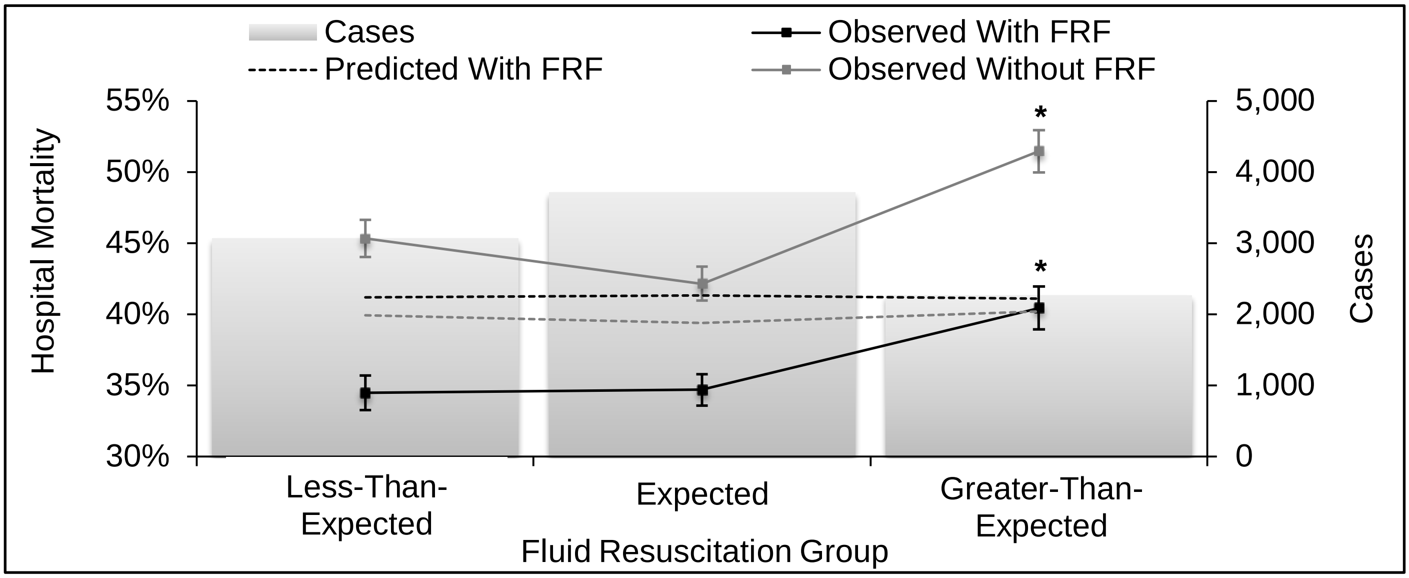

Figure E5. The effect of day one fluid volume under or over-resuscitation in patients with septic shock. Risk adjusted observed vs. predicted hospital mortality by less-than-expected ($\Delta < -1.5$L), expected ($|\Delta| < 1.5$L), or greater-than-expected ($\Delta > 1.5$L) resuscitation as determined by the difference between predicted and observed day one fluid resuscitation for septic shock patients. Risk adjustment performed by adding the predicted hospital mortality difference between expected-resuscitation and under or over-resuscitation groups to their respective observed hospital mortality.

* Indicates statistically significant difference in observed hospital mortality when compared to the expected-resuscitation group
